# Gemcitabine-Induced Thrombotic Microangiopathy in a Patient With Cholangiocarcinoma: An Atypical Case

**DOI:** 10.7759/cureus.63385

**Published:** 2024-06-28

**Authors:** Vishwanath Anil, Ensaf Alhujaily, Deeksha Grover, Jolina P Santos, Ashok Kumar Kanugula, Moyosore Suleiman, Sonu Singh

**Affiliations:** 1 Internal Medicine, Wellstar Spalding Medical Center, Griffin, USA; 2 Nephrology, Wellstar Spalding Medical Center, Griffin, USA; 3 Hematology and Medical Oncology, Wellstar Spalding Medical Center, Griffin, USA

**Keywords:** cholangiocarcinoma, end-stage renal disease (esrd), chemotherapy-related toxicity, medication adverse effect, gemcitabine-induced thrombotic microangiopathy

## Abstract

Gemcitabine-induced thrombotic microangiopathy (GITMA) is a rare but severe complication seen in cancer patients on gemcitabine therapy. This case report describes a 45-year-old female with metastatic cholangiocarcinoma on gemcitabine-capecitabine who developed acute kidney injury and hypertension without typical hematologic signs of thrombotic microangiopathy (TMA). Despite initial management targeting hypertensive urgency and acute kidney injury, renal function continued to decline and progressed to end-stage renal disease requiring hemodialysis. Laboratory tests revealed TMA features such as elevated lactate dehydrogenase (LDH), decreased haptoglobin, and schistocytes. Renal biopsy confirmed TMA with chronic features. This case highlights the challenge of diagnosing drug-induced TMA without typical hematologic findings.

## Introduction

Thrombotic microangiopathy (TMA) is an occlusive microvascular condition characterized by the predominant formation of platelet thrombi in the renal and systemic circulation. Two main clinical conditions constitute TMA: hemolytic uremic syndrome (HUS) and thrombotic thrombocytopenic purpura (TTP). HUS primarily displays kidney-related symptoms, while TTP often shows systemic and neurological manifestations. The triad of renal insufficiency, microangiopathic hemolytic anemia, and thrombocytopenia classically characterizes TMA. TMA can also lead to new-onset hypertension and other neurological or pulmonary symptoms in some instances. Though rare, TMA can lead to end-stage renal disease and may even have fatal outcomes.

Gemcitabine, a nucleoside analog, first received FDA approval in 1996 to treat metastatic pancreatic cancer. Today, it is used for various cancers such as lymphoma, as well as lung, bladder, and breast cancer. In 1994, gemcitabine therapy was linked to TMA during a pancreatic cancer trial [1]. The documented occurrence of gemcitabine-related TMA in the current literature is extremely rare, estimated by the manufacturer at 0.015% based on adverse event reports in 1997 [1]. However, reports from local institutions have revealed a higher apparent frequency of gemcitabine-associated TMA [1]. This case highlights the need to recognize drug-induced TMA that can occur without associated thrombocytopenia.

## Case presentation

A 45-year-old Caucasian female, with a past medical history of obesity, chronic kidney disease - stage 3B, hypothyroidism, obstructive sleep apnea, and metastatic cholangiocarcinoma (on chemotherapy), came into the emergency room after she was found to have elevated blood pressure (207/118 mmHg) at her oncologist’s office. Apart from some mild anxiety, the patient was otherwise asymptomatic at presentation. The patient’s chemotherapy regimen was composed of gemcitabine and capecitabine. The last dose of gemcitabine was administered about two weeks prior to this presentation. The patient was started on gemcitabine about four months prior to this presentation. Given her body surface area of 2.1 m^2^, each dose of gemcitabine was 2,000 mg. The patient received gemcitabine on day one and day eight of a 21-day cycle. She had completed five cycles.

Initial vitals showed a temperature of 97.7 F, blood pressure of 193/90 mmHg, pulse of 94 bmp, and respiratory rate of 18/min. Physical examination revealed an obese female who was in no acute distress. Conjunctival pallor was noted. A right-sided Port-A-Cath was present on the chest examination. Other physical examination findings were unremarkable. The initial blood tests reported values outlined in Table 1.

**Table 1:** Blood tests at presentation. BUN - Blood urea nitrogen, HCO3 - Bicarbonate, LDH - Lactate dehydrogenase, WBC - White blood cell

Blood Test	Result	Range
Sodium	137 mmol/L	136-145 mmol/L
Potassium	4.8 mmol/L	3.5-5.1 mmol/L
Chloride	107 mmol/L	98-107 mmol/L
HCO3	17 mmol/L	22-29 mmol/L
BUN	56 mg/dL	6-20 mg/dL
Serum Creatinine	3.73 mg/dL	0.7-1.2 mg/dL
WBC Count	8480 /µL	3500-10,500 /µL
Hemoglobin	6.2 g/dL	13.5-17.5 g/dL
Platelet Count	330,000 /µL	150,000-450,000 /µL
Absolute Reticulocyte Count	290,000 /µL	170,000-700,000 /µL
LDH	634 IU/L	98-192 IU/L
Ferritin	946 ng/mL	13-150 ng/mL

The baseline serum creatinine about six months prior to presentation was 1.85 mg/dL. The patient had a normal coagulation profile. Vitamin B12 and folate levels were within normal limits. Iron studies were unremarkable, except for an elevated ferritin level.

Urinalysis revealed protein 3+, blood 2+, and unspecified urine casts. The urine eosinophil smear was negative. The urine protein-creatinine ratio was 1,542 mg/g. A calculated fractional excretion of sodium was 0.4%, in favor of pre-renal acute kidney injury.

An ultrasound of the kidneys revealed an atrophic right kidney but no acute hydronephrosis or kidney stones. A computed tomography (CT) scan of the head at the time of admission did not reveal any acute findings.

Despite treatment with intravenous anti-hypertensives, the patient’s blood pressure remained elevated in the 160s/80s mmHg range. The patient was admitted to the hospital under the impression of acute kidney injury superimposed on chronic kidney disease - stage 3B and hypertensive urgency.

Hospital course

Given the low hemoglobin, the patient was transfused with two units of packed red blood cells. Despite the uncontrolled hypertension, the acute kidney injury was deemed to have a pre-renal component, and the patient was started on gentle IV fluids. Blood pressure was managed with both IV and PO medications.

Hematology/oncology and nephrology services were consulted for this case. The serum haptoglobin was noted to be decreased at <10 mg/dL. This further increased the suspicion of hemolytic anemia. However, the total bilirubin was unremarkable at 1 mL/dL. The differential of gemcitabine-induced thrombotic microangiopathy (GITMA) was considered despite the lack of thrombocytopenia in the picture. There were no schistocytes reported on the peripheral smear.

On day two, the serum creatinine showed a downtrend to 3.56 mg/dL, and the course of treatment was not changed. On day three, the serum creatinine up-trended to 3.91 mg/dL.

The patient’s renal function continued to decline (serum creatinine of 4.51 mg/dL) the next day despite IV fluids. On day four, the patient was noted to have symptoms of volume overload, including mild shortness of breath requiring supplemental oxygen of 2 L and mild pedal edema. This required the initiation of diuresis with IV furosemide. The patient had adequate urinary output; however, it was insufficient to resolve the volume overload completely. Given the nature of the worsening renal function, a decision was made to obtain a kidney biopsy. A biopsy sample was obtained from the left kidney under CT-image guidance. The decision was also made to place temporary dialysis access in the left internal jugular vein and initiate hemodialysis. A temporary dialysis catheter was placed on day six of admission. The patient underwent a total of three hemodialysis sessions before the decision was made to place a tunneled hemodialysis catheter. The patient underwent two more hemodialysis sessions prior to discharge. There were no signs of renal recovery in the blood work performed during this time.

**Figure 1:**
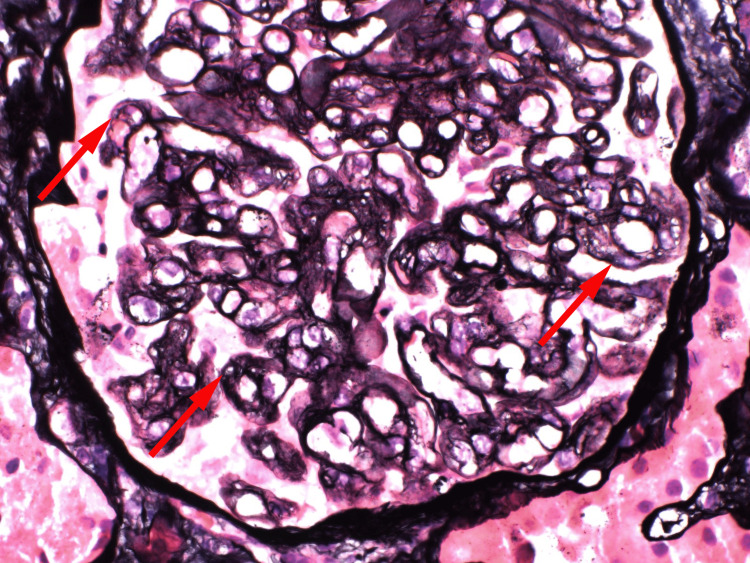
Frequent capillary loops with double contours (red arrows).

Preliminary reports of the kidney biopsy favored thrombotic microangiopathy with chronic features (glomerular basement membrane with double contours) (Figure 1), negative immunofluorescence, moderate tubular atrophy, and interstitial fibrosis. Further laboratory investigations revealed normal complement levels (C3 of 112 mg/dL and C4 of 28 mg/dL) and a mildly decreased ADAMTS13 activity level of 0.54 IU/mL. The potential benefits of eculizumab or plasmapheresis were considered in this case; however, they were not pursued due to the extent of the underlying metastasis, an average prognosis of less than 12 months, and chronicity appearing on a kidney biopsy.

After 16 days of hospitalization, the patient was discharged home with an outpatient hemodialysis schedule in place. At the time of discharge, appropriate outpatient follow-up with an oncologist and nephrologist was advised.

**Figure 2:**
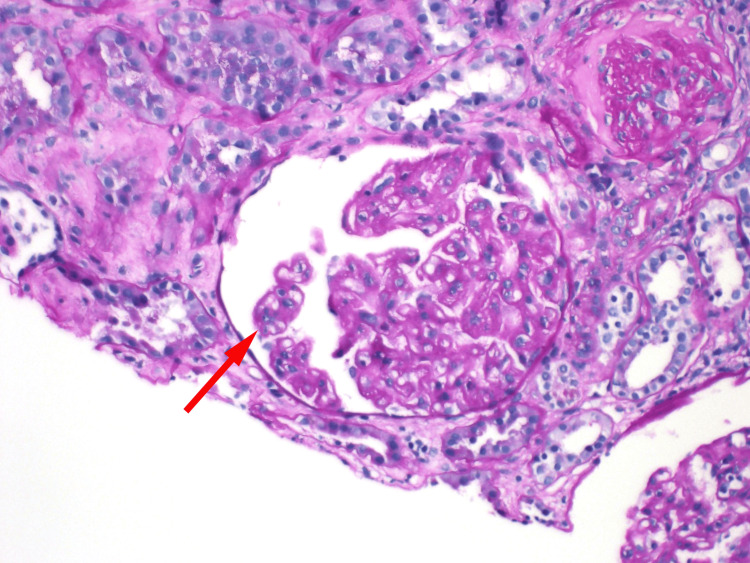
Glomerular ischemic changes with wrinkling of the capillary loops and partial collapse of the glomerular tuft (red arrow).

**Figure 3:**
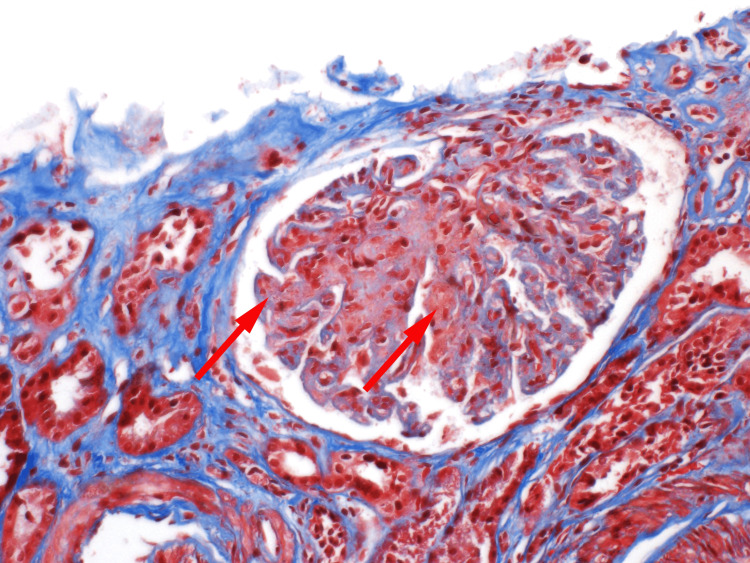
Glomerulus showing mesangiolysis (red arrow, left) and endothelial swelling (red arrow, right).

**Figure 4:**
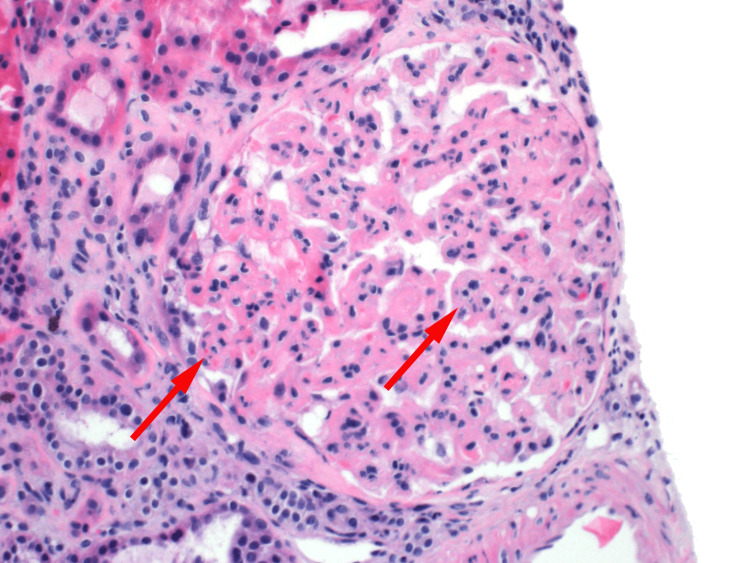
Endothelial swelling, mesangiolysis (red arrow, left), and "bloodless" appearance of a glomerulus (red arrow, right).

**Figure 5:**
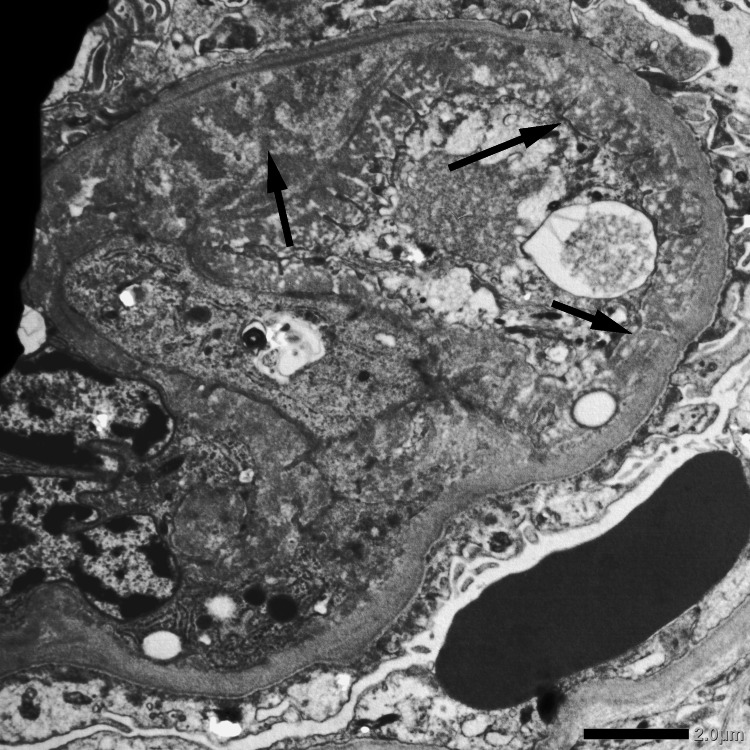
Electron micrography showing endothelial swelling (black arrow, left) and subendothelial widening (two black arrows, right).

**Figure 6:**
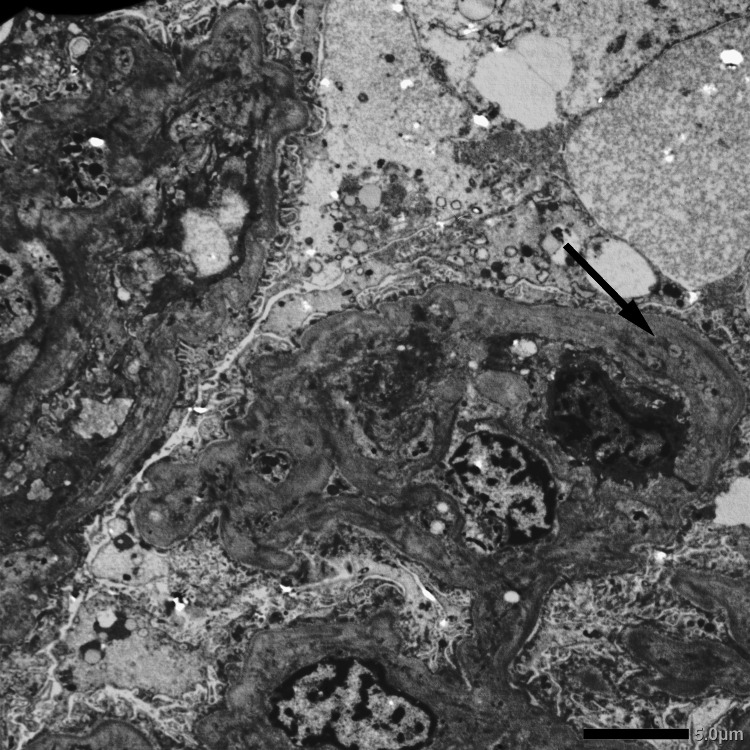
Electron micrography showing new glomerular basement membrane formation (black arrow).

The final pathology report revealed features of chronic thrombotic microangiopathy such as glomerular ischemic changes, as seen in Figures 2-4. Electron microscopy, as shown in Figures 5-6, revealed endothelial swelling and new glomerular basement membrane formation, respectively.

## Discussion

TMA is categorized as primary when its cause is unknown (idiopathic) and secondary when the symptoms arise due to various conditions, such as cytotoxic chemotherapy, infection, autoimmune disorders, pregnancy, malignancy, or bone marrow transplantation [2]. TMA in a patient with cancer can stem from either the cancer itself or drug-related factors [3].

In the clinical setting, TMA presents as either HUS or TTP [4]. Chemotherapy-induced TMA often exhibits similarities to atypical HUS (aHUS) [5]. While HUS and TTP are considered rare within the general population, the incidence of TMA among cancer patients ranges from 6% to 15%, escalating to around 40% in the context of hematopoietic stem cell transplants (HSCT) [5]. Secondary TMAs constitute most of the cases (80%-90%) [5,6]. TMA has been reported to be induced by various anticancer medications, including gemcitabine, ramucirumab, docetaxel, and nivolumab [7]. A seven-year French investigation involving 564 hospitalized patients managing TMA revealed that the majority (94%) of cases were linked to secondary causes. Among these secondary cases, malignancies accounted for 19% of the causes, while drugs accounted for 26%. The drugs implicated included calcineurin inhibitors (68%), gemcitabine (8%), and vascular endothelial growth factor inhibitors (3%) [5].

The specific mechanisms behind gemcitabine-induced TMA are not fully understood [3]. Gemcitabine is a nucleoside analog closely related to cytarabine. It has been used in chemotherapy regimens across multiple malignancies, such as pancreatic and non-small cell lung cancer, as well as urothelial and ovarian cancers [8].

A disintegrin and metalloproteinase with a thrombospondin type 1 motif 13 (ADAMTS13) is demonstrated to be significant in the pathophysiology of TTP when either antibodies against ADAMTS13 are present (acquired TTP) or when antibodies against ADAMTS13 are lacking (inherited TTP). A severe deficiency of ADAMTS‐1 activity, with clinical symptoms of acute thrombocytopenia and evidence of microangiopathic hemolytic anemia, constitutes a diagnosis of TTP [9]. HUS is not associated with a deficiency in ADAMTS13 activity [9].

Recent research, based on a retrospective study, indicates the presence of C5b9 deposits in kidney biopsies, suggesting that complement activation, potentially triggered by the drug's direct harm to endothelial cells, might play a partial role [3]. This could clarify why using eculizumab as a treatment might show promise in these cases. Besides drug-induced TMA (DITMA) caused by direct harm to endothelial cells (type 1, dosage-dependent) or immune responses, resulting in drug-triggered autoantibodies (type 2, independent of dosage), the malignancy itself can lead to TMA (paraneoplastic process). This can occur from metastases to the microvasculature or the infiltration of tumor cells into the bone marrow [3].

Patients with TMA might display increasing creatinine levels, new or worsening hypertension, proteinuria, and edema. Reports indicate that 75%-90% of individuals experiencing chemotherapy-associated TMA also manifest hypertension [1]. Proteinuria tends to be sub-nephrotic, but it can occasionally reach nephrotic levels (>3 g/d) [5]. Signs of renal involvement in DITMA can vary significantly. They might manifest as a moderate rise in serum creatinine following mild proteinuria and microscopic hematuria. Alternatively, they can present dramatically as acute oliguric or anuric renal failure, often accompanied by salt and water retention [1]. In our case, it was noted that the patient started to insidiously develop hypertension over the last two to three months prior to this presentation. The patient was notably in hypertensive urgency at the time of presentation. The new onset of hypertension would serve as a clinical indicator for worsening renal function in a patient receiving gemcitabine.

Laboratory assessments should encompass a complete blood count with differential, LDH, haptoglobin, and a peripheral smear to detect schistocytes. However, it is essential to note that hematologic features characteristic of TMA might only sometimes be evident in DITMA. Reduced haptoglobin levels occur due to binding to free hemoglobin, subsequently cleared by macrophages. Elevated LDH levels are expected due to tissue ischemia and cellular breakdown. The negative Coombs test aligns with intravascular hemolysis. Schistocytes, indicative of red blood cell fragmentation within the microvasculature, are considered significant at a count of more than 1% or 2% per high-power field [5].

Coagulation tests typically remain within the normal range, distinguishing it from conditions such as disseminated intravascular coagulation (DIC), which warrants consideration in patients with an active malignancy [5]. The coagulation profile remained within normal limits in our case. However, the normal platelet count was an atypical finding, which made the diagnosis more challenging. An essential step in diagnosis involves excluding TTP by assessing ADAMTS13 activity. In specific clinical scenarios, evaluations of vitamin B12, homocysteine levels, methylmalonic acid, and Shiga toxin and Escherichia coli analysis in cases presenting with diarrhea should also be pursued to assist in accurate diagnosis [5].

Theoretically, a definitive diagnosis depends on a renal biopsy, in which glomerular and/or arteriolar fibrin deposits are demonstrated using the immunofluorescence technique [1]. However, the literature supports that renal biopsies are only routinely indicated in these cases if the symptoms are unusual. The clinical and lab results often strongly suggest an HUS or TTP picture. Renal biopsies do not usually change the management of these conditions. Biopsies are also often risky, given the thrombocytopenia and other comorbidities in these patients. The typical biopsy findings include histopathological features such as basement membrane duplication, mesangiolysis, tubular injury with interstitial fibrosis, and glomerular endothelial cell swelling [10].

According to Humphreys et al., the onset or exacerbation of hypertension could potentially serve as an early indicator for identifying the progression of a TMA syndrome before other symptoms manifest. Detecting TMA in patients undergoing gemcitabine treatment poses a challenge, as common chemotherapy-related effects such as anemia and thrombocytopenia may not reliably signal its presence. The suggestion is that a deterioration in thrombocytopenia or anemia, coupled with newly heightened hypertension and renal insufficiency, should prompt clinicians to investigate for signs of microangiopathic hemolysis [4]. With a large retrospective cohort study, Daviet et al. concluded that systematic screening for hypertension and/or edema in patients treated with gemcitabine would help early TMA detection. The control of hypertension and close monitoring to determine the optimal timing for gemcitabine cessation could prevent full-blown drug-induced thrombotic microangiopathy (DITMA) and severe outcomes [11].

The most appropriate management of DITMA primarily involves stopping the suspected medication and providing supportive care. When the medication is withdrawn or its dosage reduced, improvement or resolution of TMA is often observed. However, merely discontinuing the drug may not always lead to complete clinical recovery, and some level of kidney damage may persist. For such patients, particularly those with advanced kidney disease, additional treatments may be necessary. The limited understanding of the underlying causes of DITMA and the lack of clinical trials in this area are reflected in treatment guidelines not strongly supported by scientific evidence [6].

Eculizumab, functioning as a terminal complement inhibitor, is a humanized monoclonal antibody targeting the human C5 complement protein. It works by binding strongly to C5, effectively obstructing the formation of proinflammatory substances such as C5a and C5b-9. Clinical trials have documented that eculizumab improved thrombocytopenia and demonstrated a consistent, progressive enhancement in the estimated glomerular filtration rate (GFR) over time. Additionally, it has been linked to improving health-related quality of life for individuals receiving treatment [8]. Rituximab, an anti-CD20 monoclonal antibody, is yet another therapeutic option that has reportedly been used in GITMA with some positive results [5].

Therapeutic plasma exchange (TPE) is a treatment modality often employed in TMA. The purpose of TPE in the management of TMA is to remove von Willebrand factor (vWF) multimers, replenish ADAMTS13, and remove any other potential autoantibodies. TPE has significantly improved mortality from TTP, decreasing it to less than 20%. However, in chemotherapy-associated TMA, it has not been shown to improve outcomes. The American Society for Apheresis guidelines have classified TPE for chemotherapy (gemcitabine) as ineffective (category IV) [5].

In a significant French retrospective study involving 120 patients with gemcitabine-induced TMA (GITMA), it was found that individuals treated with plasma exchange (PE) showed similar remission rates compared to those who did not undergo PE [12]. However, the PE-treated group experienced a higher occurrence of adverse events [12]. The prognosis for GITMA is concerning, as nearly half of the patients advance to end-stage kidney disease, accompanied by a high mortality rate ranging between 40% and 90% [12].

## Conclusions

This case focuses on a rare case of GITMA without thrombocytopenia in a patient with cholangiocarcinoma. DITMA stands as one of the most prevalent manifestations of TMA and poses a potential threat to life. The unclear mechanisms responsible for drug-induced endothelial damage contribute to its complexity. While genetic variations in complement genes are seldom found in DITMA patients, more than half show acquired complement hyperactivation systemically or in the kidneys. The rapid potential reversal of pathological indicators in DITMA emphasizes the crucial need for early suspicion, identification, and cessation of the suspected medication to minimize organ dysfunction. In severe instances of DITMA unresponsive to discontinuing the drug, treatments such as therapeutic plasma exchange, rituximab, or eculizumab may be employed as last-resort therapies. Further research is imperative to comprehend the pathophysiology and appropriate treatment guidelines in cases of DITMA. In addition, this case study aligns with others in warranting regular monitoring of renal function during gemcitabine treatment.
